# The genus *Castanediella*

**DOI:** 10.3897/mycokeys.51.32272

**Published:** 2019-04-15

**Authors:** Chuan-Gen Lin, Darbhe J. Bhat, Jian-Kui Liu, Kevin D. Hyde, Yong Wang

**Affiliations:** 1 Department of Plant Pathology, College of Agriculture, Guizhou University, Guiyang, Guizhou 550025, China Mae Fah Luang University Chiang Rai Thailand; 2 Center of Excellence in Fungal Research, Mae Fah Luang University, Chiang Rai 57100, Thailand Guizhou University Guiyang China; 3 128/1-J, Azad Housing Society, Curca, Goa Velha 403108, India Azad Housing Society Goa Velha India; 4 Formerly, Department of Botany, Goa University, Goa, India Goa University Goa India; 5 Center for Bioinformatics, School of Life Science and Technology, University of Electronic Science and Technology of China, Chengdu 611731, China University of Electronic Science and Technology of China Chengdu China

**Keywords:** new taxa, Castanediellaceae, hyphomycetes, phylogeny, Sordariomycetes

## Abstract

Two new species, *Castanediellabrevis* and *C.monoseptata*, are described, illustrated and compared with other *Castanediella* taxa. Evidence for the new species is provided by morphological comparison and sequence data analyses. *Castanediellabrevis* can be distinguished from other *Castanediella* species by the short hyaline conidiophores and fusiform, aseptate hyaline conidia, while *C.monoseptata* differs from other *Castanediella* species by its unbranched conidiophores and fusiform, curved, 0–1-sepatate, hyaline conidia. Phylogenetic analysis of combined ITS and LSU sequence data was carried out to determine the phylogenetic placement of the species. A synopsis of hitherto described *Castanediella* species is provided. In addition, *Castanediella* is also compared with morphologically similar-looking genera such as *Idriella*, *Idriellopsis*, *Microdochium*, *Neoidriella*, *Paraidriella* and *Selenodriella*.

## Introduction

Hernández-Restrepo et al. (2017) introduced the family *Castanediellaceae* for the genus *Castanediella* within Xylariales and it was consolidated in recent study by Wijayawardene et al. (2018). The asexual morphs in *Castanediellaceae* are hyphomycetous and characterized by macronematous, mononematous or sporodochial, branched, brown to pale brown conidiophores, with monoblastic or polyblastic, sympodial, discrete, cylindrical to lageniform, hyaline to subhyaline conidiogenous cells, that produce unicellular or transversely septate, cylindrical, fusiform or lunate, hyaline conidia (Hernández-Restrepo et al. 2017).

The genus *Castanediella* was established by Crous et al. (2015) to accommodate *C.acaciae*, *C.cagnizarii* and *C.ramosa* within *Xylariales* genera *incertae sedis*. The genus contains twelve species (Costa et al. 2018; Wanasinghe et al. 2018), each characterized by branched, hyaline to pale brown conidiophores, holoblastic, sympodial conidiogenous cells and falcate, cylindrical or fusiform, 0–3-sepate, hyaline conidia (Crous et al. 2015; Costa et al. 2018).

During a survey of hyphomycetes in Thailand, two hyaline-spored hyphomycetes were collected. They were shown to belong to the genus *Castanediella* based on morphology and phylogeny analyses of ITS and LSU sequence data. The new species *C.brevis* and *C.monoseptata* are introduced.

## Materials and methods

### Collection and isolation of fungi

Dead leaves from a variety of plants in two forests (Lampang province and Chiang Mai province) were collected in 2016 in Thailand. Samples were taken to the laboratory in Zip-lock plastic bags for examination. The specimens were incubated in sterile moist chambers and examined using a Motic SMZ 168 series microscope. Fungi were removed with a needle and placed in a drop of distilled water on a slide for morphological study. Photomicrographs of fungal structures were captured with a Canon 600D digital camera attached to a Nikon ECLIPSE Ni compound microscope. All measurements were made by the Tarosoft (R) Image FrameWork program. Photo-plates were made with Adobe Photoshop CS3 (Adobe Systems, USA). Isolation of the fungi on to potato dextrose agar (PDA) was performed by the single spore isolation method (Chomnunti et al. 2014). Dried material was deposited in the Herbarium of Mae Fah Luang University (**MFLU**), Chiang Rai, Thailand and herbarium of Kunming Institute of Botany, Chinese Academy of Sciences (**HKAS**), Kunming, China. Cultures were deposited at Mae Fah Luang University Culture Collection (**MFLUCC**), Chiang Rai, Thailand and Kunming Institute of Botany, Chinese Academy of Sciences (**KUMCC**), Kunming, China. FacesofFungi and Index Fungorum numbers were registered (Jayasiri et al. 2015; Index Fungorum 2018).

### DNA extraction, PCR amplification and sequencing

Genomic DNA was extracted from fungal mycelium grown on PDA or malt extract agar (MEA) at room temperature using the Fungal gDNA Kit (BioMIGA, USA) according to the manufacturer’s instructions. The internal transcribed spacer region of ribosomal DNA (ITS) and large subunit nuclear ribosomal DNA (LSU) genes were amplified via polymerase chain reaction (PCR) using the following primers: ITS5 and ITS4 (White et al. 1990) for ITS, and LR0R and LR5 (Vilgalys and Hester 1990) for LSU. The PCR products were sequenced with the same primers. The PCR amplification was performed in a 25 μL reaction volume containing 12.5 μL of 2 × Power Taq PCR MasterMix (a premix and ready to use solution, including 0.1 Units/μl Taq DNA Polymerase, 500 μM dNTP Mixture each [dATP, dCTP, dGTP, dTTP], 20 mM Tris-HCl pH 8.3, 100 Mm KCl, 3 mM MgCl_2_, stabilizer and enhancer), 1 μL of each primer (10 μM), 1 μL genomic DNA extract and 9.5 μL deionised water. The PCR thermal cycle program of ITS and LSU were followed as: initially 94 °C for 3 min., followed by 35 cycles of denaturation at 94 °C for 30 s, annealing at 55 °C for 50 s, elongation at 72 °C for 1 min., and final extension at 72 °C for 10 min.

### Phylogenetic analyses

Original sequences were checked using BioEdit version 7.0.5.3 (Hall 1999), and most reference sequences were originated from previous publications. The remaining homogenous sequences were obtained by BLAST searches (Altschul et al. 1990) from GenBank. All sequences used in this study are listed in Table 1. Alignments for each locus were done in MAFFT v7.307 online version (Katoh and Standley 2016) and manually verified in MEGA 6.06 (Tamura et al. 2013). After alignment, the concatenation of different genes was done in SequenceMatrix 1.8 (Vaidya et al. 2011). The interleaved NEXUS files for Bayesian inference analyses were formatted with AliView v1.19-beta1k (Larsson 2014). Maximum parsimony (MP), maximum likelihood (ML) and Bayesian inference (BI) were used for phylogenetic analyses.

**Table 1.** GenBank accession numbers of isolates included in this study.

Taxa	Isolate^a^	ITS	LSU
* Castanediella acaciae *	CPC 24869, CBS 139896	NR_137985	KR476763
*Castanediellabrevi*s	KUMCC 18-0132	MH806361	MH806358
* Castanediella cagnizarii *	MUCL 41095	KC775732	KC775707
* Castanediella cagnizarii *	CBS 101043	KP859051	KP858988
* Castanediella cagnizarii *	CBS 542.96	KP859054	KP858991
* Castanediella camelliae *	CNUFC-DLHBS5-1	MF926620	MF926614
* Castanediella camelliae *	CNUFC-DLHBS5-2	MF926621	MF926615
* Castanediella communis *	CPC 27631	KY173393	–
* Castanediella couratarii *	CBS 579.71	NR_145250	KP858987
* Castanediella eucalypti *	CPC 24746, CBS 139897	NR_137981	KR476758
* Castanediella eucalypticola *	CPC 26539	NR_145254	KX228317
* Castanediella eucalyptigena *	CBS 143178, CPC 32055	MG386036	MG386089
* Castanediella hyalopenicillata *	CPC 25873	KX306751	KX306780
* Castanediella malaysiana *	CPC 24918	NR_154810	KX306781
*Castanediellamonoseptat*a	KUMCC 18-0133	MH806360	MH806357
* Castanediella ramosa *	MUCL 39857	KC775736	KC775711
* Subsessila turbinata *	MFLUCC 15-0831	KX762288	KX762289

^a^**CBS**, Centraalbureau voor Schimmelcultures, Utrecht, Netherlands; **CPC**, Culture collection of Pedro Crous, housed at CBS; **KUMCC**, Kunming Institute of Botany, Chinese Academy of Sciences, Kunming, China; **MFLUCC**, Mae Fah Luang University Culture Collection, Chiang Rai, Thailand; **MUCL**, Mycothèque de l’Université Catholique de Louvian, Belgium.

The best models of evolution for each gene region were determined using Akaike information criterion (AIC) as implemented in MrModeltest v2 (Nylander 2004). The analyses’ results showed that the models GTR+I and GTR+I+G were the best ones for LSU and ITS sequence data, respectively.

MP analyses were performed in PAUP*4.0b10 (Swofford 2002) following Liu et al. (2016).

ML analyses were carried out in raxmlGUI v 1.5b1 (Silvestro and Michalak 2012) with RAxML v8.2.10 (Stamatakis 2014), using the ML + rapid bootstrap setting and the GTRGAMMAI (viz., GTR + GAMMA + I) substitution model with 1000 bootstrap replicates.

For BI analysis, Posterior probabilities (PP) (Rannala and Yang 1996; Zhaxybayeva and Gogarten 2002) were determined by Markov Chain Monte Carlo sampling (BMCMC) in MrBayes v 3.2.6 (Ronquist et al. 2012). For the combined dataset, the models were set to nst = 6 and rates = propinv for LSU and nst = 6 and rates = invgamma for ITS. Two independent analyses of two parallel runs and six simultaneous Markov chains were run for 1,000,000 generations, trees were sampled every 100^th^ generation and the temperature value of the heated chains was set at 0.15. The first 25% sampled trees of each run were discarded as “burn-in”, and the remaining trees were used for calculating posterior probabilities (PP) in the majority rule consensus tree with the sumt command in MrBayes.

Phylogenetic trees were drawn with TreeView 1.6.6 (Page 1996).

## Results

### Molecular phylogeny

**Figure 1.**
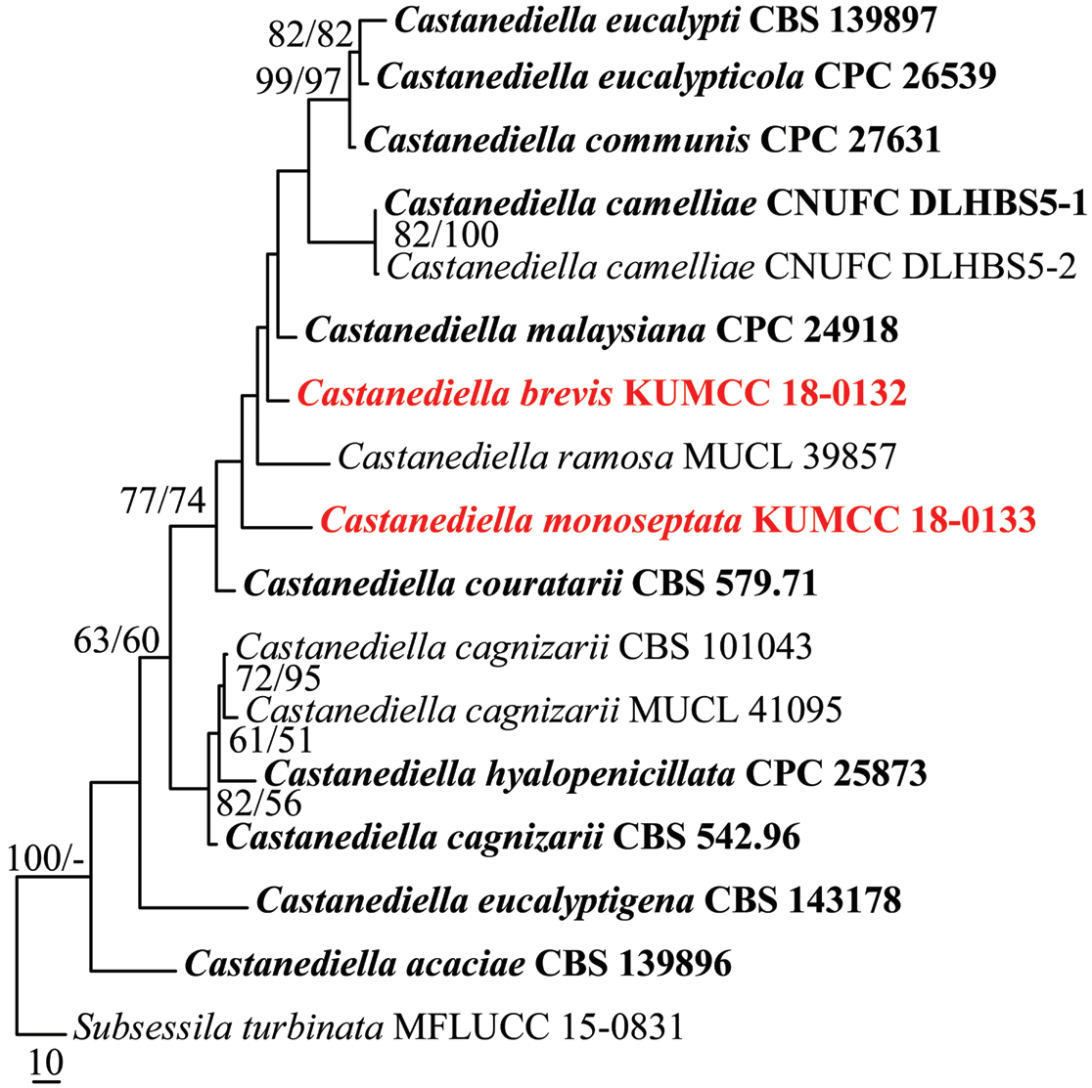
Phylogenetic tree generated from MP analysis based on combined LSU and ITS sequence data for the genus *Castanediella*. Bootstrap support values for maximum parsimony (MP, first set) and maximum likelihood (ML, second set) greater than 50% are indicated above or below the nodes. Ex-type strains are in bold, the new isolates are in red. The tree is rooted with *Subsessilaturbinata* (MFLUCC 15-0831).

The aligned sequence matrix comprises LSU and ITS sequence data for 16 taxa (ingroup) and one outgroup taxon with a total of 1438 characters after alignment including the gaps, of which 120 were parsimony informative, 77 parsimony-uninformative, and 1241 characters constant. The dataset consists of thirteen species within the genus. The tree was rooted with *Subsessilaturbinata* (MFLUCC 15-0831). Maximum parsimony analysis resulted in two trees with TL = 391, CI = 0.657, RI = 0.642, RC = 0.422, HI = 0.343. For the Bayesian analysis, two parallel runs with six chains were run for 1,000,000 generations and trees were sampled every 100^th^ generation, resulting in 20002 trees from two runs of which 15002 trees were used to calculate the posterior probabilities (each run resulted in 10001 trees of which 7501 trees were sampled).The MP and ML (lnL = -4041.301739) analyses based on combined LSU and ITS sequence data provided similar tree topologies, and the result of MP analysis is shown in Fig. 1.

The novelty of the species, *Castanediellabrevis* and *C.monoseptata*, described in this study are supported by sequence data analyses as belonging to the genus *Castanediella*, but with low bootstrap support values. Isolates of *Castanediellabrevis* and *C.monoseptata* formed separate clades in the phylogenetic inference, respectively. *Castanediellabrevis* is sister to *C.malaysiana* and *C.ramosa*, while *C.monoseptata* shows close phylogenetic relationship to *C.couratarii* and *C.malaysiana*. Both the new taxa can be recognized as phylogenetically distinct species and are clearly novel based on the recommendations for molecular data (Jeewon and Hyde 2016).

MP, ML and BI were used for phylogenetic analyses in this study. The tree topologies of MP and ML resulted from the combined LSU and ITS sequence data are similar, but most of the nodes are in low bootstrap support (Fig. 1). Polytomy structure was formed in the BI tree generated from the combined LSU and ITS sequence data. More sequence data, especially the protein-coding genes, e.g. TEF1-α, RPB2, β-tubulin, are required in the future study of the genus *Castanediella*.

### Taxonomy

#### Castanediella brevis

Taxon classificationFungiXylarialesCastanediellaceae

s C.G. Lin & K.D. Hyde sp. nov.

MB828879

Figure 2

##### Holotype.

THAILAND. Lampang: Amphoe Mueang Pan, Tambon Chae Son, on decaying leaves, 24 September 2016, Chuangen Lin, LCG 10-1 (MFLU 18-1695, holotype; HKAS 102198, isotype), ex-type living cultures KUMCC 18-0132.

##### GenBank number.

ITS: MH806361, LSU:MH806358

##### Etymology.

In reference to the short conidiophores.

*Saprobic* on plant host. **Asexual morph**: *Colonies* on substrate effuse, white. *Mycelium* partly superficial, composed of septate, branched, smooth, hyaline to subhyaline hyphae. *Conidiophores* macronematous, mononematous, solitary, erect, unbranched, straight or flexuous, short, 0–1-septate, hyaline, subcylindrical, ampulliform, smooth, often reduced to conidiogenous cells. *Conidiogenous cells* holoblastic, polyblastic, sympodial, integrated, terminal, subcylindrical, ampulliform, hyaline, denticulate, with 2–4 tiny protuberant denticles, 3–14 × 1.5–5.5 μm. *Conidia* solitary, dry, acropleurogenous, smooth, fusiform, curved, aseptate, hyaline, 12.5–21.7 × 1.2–3 μm (av. 16.95 × 2.2 μm, n = 60). **Sexual morph**: Undetermined.

*Culture characteristics*: Conidia germinating on PDA within 24 h. Colonies on PDA effuse, greyish white to dark from above and below, reaching a diam. of 5–7 cm in 30 days at 25 °C.

##### Notes.

Based on a megablast search of the NCBI nucleotide database using the ITS sequence of the ex-type culture, the highest similarities found were with *Castanediellamalaysiana* (GenBank NR_154810; identities = 526/537(98%), gaps = 1/537(0%)) and *C.couratarii* (GenBank KX960789; identities = 521/538(97%), gaps = 3/538(0%)). *Castanediellabrevis* differs from these two species by its conidiophore morphology. *Castanediellacouratarii* has pale brown conidiophores and longer conidiogenous cells (10.5–37 × 2–3.5 μm) whereas *C.malysiana* has pale brown and longer conidiophores (76–157 × 2.5–3 μm).

Among the species that produce more or less falcate and aseptate conidia, *Castanediellacommunis*, *C.eucalypti*, *C.eucalypticola* and *C.eucalyptigena* are most similar to *C.brevis*. However, *Castanediellabrevis* differs from these species by its short, unbranched and 0–1-septate conidiophores.

**Figure 2.**
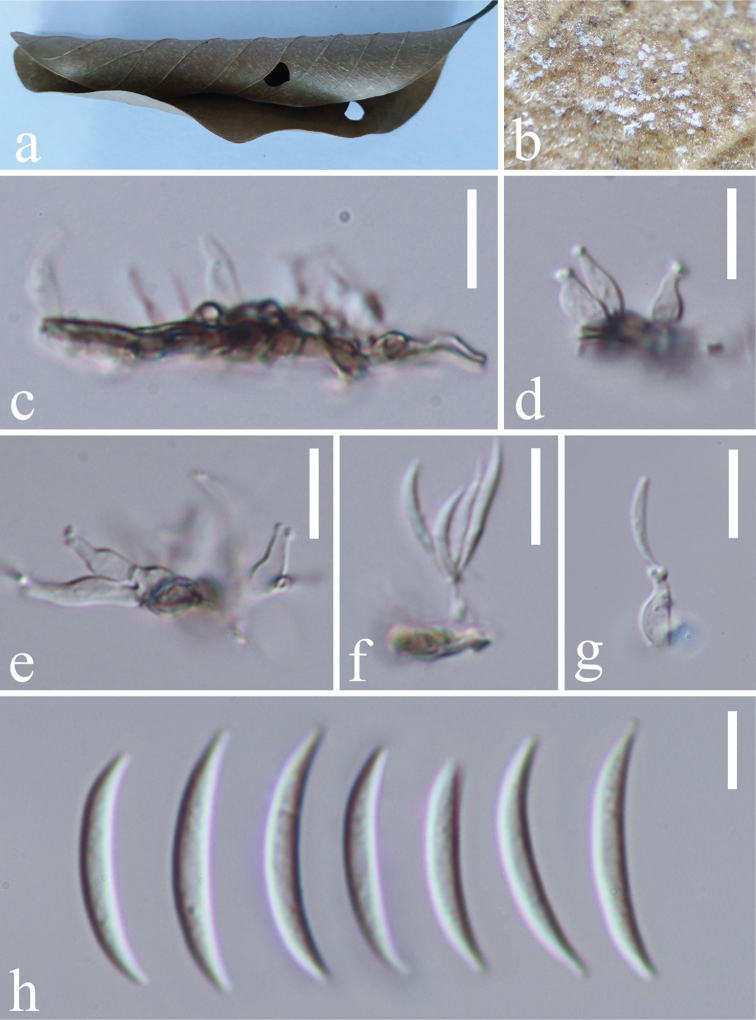
*Castanediellabrevis* (MFLU 18-1695, holotype) **a** host material **b** conidiophores on the host surface **c–g** conidiophores, conidiogenous cells with conidia **h** conidia. Scale bars: 10 μm (**c–g**), 5 μm (**h**).

#### Castanediella monoseptata

Taxon classificationFungiXylarialesCastanediellaceae

C.G. Lin & K.D. Hyde sp. nov.

MB828881

Figure 3

##### Holotype.

THAILAND. Chiang Mai: on decaying leaves, 24 August 2016, Chuangen Lin, MRC 3-1 (MFLU 18-1696, holotype; HKAS 102199, isotype), ex-type living cultures KUMCC 18-0133.

##### GenBank number.

ITS: MH806360, LSU: MH806357

##### Etymology.

In reference to the 0–1-septate conidia

*Saprobic* on plant host. **Asexual morph**: *Colonies* on substrate effuse, white. *Mycelium* partly superficial, composed of septate, branched, hyaline to subhyaline, smooth hyphae. *Conidiophores* macronematous, mononematous, solitary, erect, unbranched, straight or flexuous, septate, hyaline, subcylindrical, smooth, 8–29 × 2–4 μm. *Conidiogenous cells* polyblastic, integrated, sympodial, subcylindrical, hyaline, with several scars. *Conidia* solitary, dry, acropleurogenous, smooth, fusiform, curved, 0–1-sepatate, hyaline, 15.4–25.8 × 1.5–2.3 μm (av. 23.03 × 1.98 μm, n = 45). **Sexual morph**: Undetermined.

*Culture characteristics*: Conidia germinating on PDA within 24 h. Colonies on PDA effuse, grayish white to dark from above and below, reaching a diam. of 5–7 cm in 30 days at 25 °C.

##### Notes.

A megablast search of the NCBI nucleotide database using the ITS sequence of the ex-type culture showed the highest similarities with uncultured Sordariales fungi (GenBank GQ268569; identities = 518/539(96%), gaps = 3/539(0%)) and *Castanediellacouratarii* (GenBank KX960789; identities = 516/540(96%), gaps = 4/540(0%)).

Five *Castanediella* species, *C.cagnizarii*, *C.diversispora*, *C.hyalopenicillata*, *C.malaysiana* and *C.ramosa*, were reported to produce 1-septate conidia. *Castanediellamonoseptata* can be distinguished from these species by its unbranched conidiophores and falcate and 15.4–25.8 × 1.5–2.3 μm conidia. *Castanediellamonoseptata* is phylogenetically closely related to *C.couratarii* and *C.ramosa*, but differs from both species by its conidial morphology. *Castanediellacouratarii* has shorter conidia (9.5–19 × 2–3 μm) are aseptate and *C.ramosa* has larger conidia (26–44 × 2–3 μm) that are 0–3-septate.

**Figure 3.**
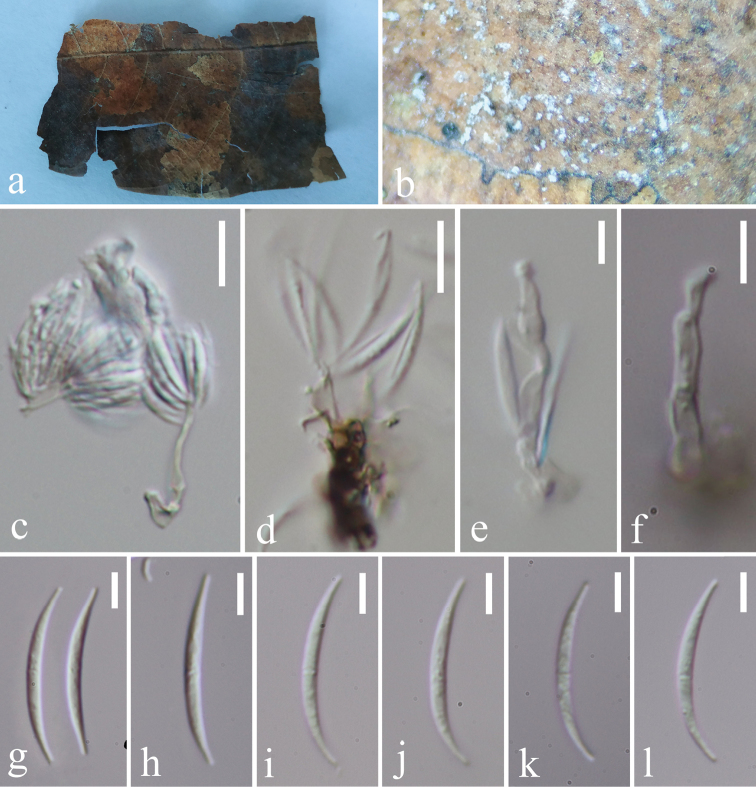
*Castanediellamonoseptata* (MFLU 18-1696, holotype) **a** host material **b** conidiophores on the host surface **c–f** conidiophores, conidiogenous cells with conidia **g–l** conidia. Scale bars: 10 μm (**c, d**), 5 μm (**e–l**).

## Discussion

In this study, two new *Castanediella* species, *C.brevis* and *C.monoseptata*, were identified from decaying leaves in Thailand and a synopsis of hitherto described *Castanediella* species is provided (Table 2).

**Table 2.** Synopsis of *Castanediella* species.

Taxa	Conidiophores	Conidiogenous cells	Conidia			
			Shape	Size (μm)	Septa	Colour
* C. acaciae *	Subcylindrical, medium brown, 40–80 × 2–3 μm.	Polyblastic, ampulliform, pale brown, 10–15 × 2–3 μm.	Falcate with subobtuse ends	(8–)10–11(–12) × 1.5(–2)	0	Hyaline
* C. brevis *	Subcylindrical, ampulliform, hyaline, often reduced to conidiogenous cells	Polyblastic, cylindrical, hyaline, 3–14 × 1.5–5.5 μm	Fusiform, curved	12.5–21.7 × 1.2–3.0	0	Hyaline
* C. cagnizarii *	Cylindrical, brown at the base, subhyaline towards the apex, up to 45 μm long.	Polyblastic, sympodial, subhyaline, 5–22 × 3–4 μm.	Cylindrical to fusiform, curved at the ends	Two sizes, 10–15 × 2 or 20–26 × 2		Hyaline
* C. camelliae *	Conidiophores reduced to conidiogenous cell.	Cylindrical, ampulliform, globose to subglobose, or irregularly-shaped, 5.5–20.5 × 2–4.5 μm.	Straight to slightly curved, sometimes swollen in the middle part	18.5–51.5 × 1.6–2.5	Septum indistinct	Hyaline
* C. communis *	Subcylindrical, medium brown, 20–60 × 3–4 μm.	Polyblastic, subcylindrical to ampulliform, pale brown, 10–35 × 2–4 μm.	Falcate with subobtuse ends	(13–)17–20(–22) × (2–)2.5(–3)	0	Hyaline
* C. couratarii *	Pale brown	Lageniform to cylindrical, hyaline to pale brown, 10.5–37 × 2–3.5 μm	Lunate	9.5–19 × 2–3	0	Hyaline
* C. diversispora *	Pale brown to brown	Polyblastic, sympodial, pale brown to brown, 4–9 × 2–3.5 μm.	Type i) cylindrical, slightly uncinate at the ends, straight	Type i) 11.5–16 × 2	Type i) 1-septate	Hyaline
			Type ii) cylindrical to slightly subacerose, slightly uncinate at the apex, abruptly attenuated at the base, straight	Type ii) 19.5–25 × 1.5–2	Type ii) 1-septate	
			Type iii) long filiform, obtuse or rounded at the apex attenuated at the base, straight or curved	Type iii) 28.5–47 × 1	Type iii) 1–3-septate	
* C. eucalypti *	Subcylindrical, medium brown, 10–30 × 3–4 μm.	Polyblastic, subcylindrical to ampulliform, pale brown, 8–25 × 2.5–4 μm.	Falcate, slightly curved, widest in middle with subobtuse ends	(15–)18–21(–23) × 2–3	0	Hyaline
* C. eucalypticola *	Subcylindrical, medium brown, 5–30 × 3–5 μm.	Polyblastic, subcylindrical to ampulliform or lanceolate, pale brown, 5–20 × 3–3.5 μm.	Falcate, straight to curved, widest in the middle, apex subobtusely rounded, base truncate, 0.5 μm diam	(15–)20–26(–30) × (2.5–)3	0	Hyaline
* C. eucalyptigena *	Subcylindrical, hyaline, frequently reduced to conidiogenous loci on hyphae, up to 15 μm tall, 3–5 μm diam.	Polyblastic, hyaline, ampulliform or subcylindrical, 2–10 × 2–5 μm	Falcate, tapering to acute ends that are subobtusely rounded	(13–)18–24(–30) × 2(–2.5)	0	Hyaline
* C. hyalopenicillata *	Cylindrical, penicillate, mono-, bi-, and terverticillate, hyaline, 24–69 × 1.5–3 μm.	Mono- and polyblastic, short cylindrical, ampulliform, hyaline, 6.5–14 × 2–4 μm	Fusiform, base pointed, apex obtuse	14–24 × 2–3	0–1	Hyaline
* C. malaysiana *	Cylindrical, biverticillate, pale brown, 76–157 × 2.5–3 μm.	Polyblastic, cylindrical, subcylindrical, hyaline, 19–28 × 2.5–3.5 μm.	Fusiform, curved, apex acuminate, and base acuminate or slightly flattened	18–30 × 2–3	0–1	Hyaline
* C. monoseptata *	Subcylindrical, unbranched, hyaline, 8–29 × 2–4 μm	Polyblastic, cylindrical, hyaline	Fusiform, curved	15.4–25.8 × 1.5–2.3	0–1	Hyaline
* C. ramosa *	Cylindrical, penicillate, brown at the base, subhyaline at the apex, up to 70 μm long	Polyblastic, subhyaline, 10–20 x 2.5–3.5 μm	Falcate	26–44 × 2.2–3	(0–) 1 (–3)	Hyaline

Presently, the genus *Castanediella* contains 14 species, and is shown to be diverse in its habitats. Most of *Castanediella* species have been collected from plant leaves. *Castanediellaacaciae*, *C.camelliae*, *C.communis*, *C.eucalypti*, and *C.eucalypticola* were isolated from disease symptoms on different host plant leaves (Crous et al. 2015, 2016a, b; Wanasinghe et al. 2018) whereas *C.cagnizarii* is the only species found on decaying leaves submerged in a stream (Castañeda Ruiz et al. 2005). Some *Castanediella* species were reported from decaying leaves, such as *C.brevis*, *C.cagnizarii*, *C.diversispora*, *C.hyalopenicillata* and *C.monoseptata* (Castañeda Ruiz et al. 2005; Hernández-Restrepo et al. 2016b; Costa et al. 2018). *Castanediellacouratarii* was reported from dead wood (Hernández-Restrepo et al. 2016a).

The genus *Castanediella* is morphologically similar to *Idriella*, *Idriellopsis*, *Microdochium*, *Neoidriella*, *Paraidriella*, *Selenodriella* (Seifert et al. 2011; Crous et al. 2015; Hernández-Restrepo et al. 2016a). However, these genera can be distinguished by the branching pattern of their conidiophores and conidial shape and septation (Hernández-Restrepo et al. 2016a). *Castanediella* differs from these genera by its branched conidiophores, ampulliform conidiogenous cells with scars instead of denticles, and filiform, 0–1-septate, straight to curved conidia (Crous et al. 2015). These similar-looking genera are phylogenetically distinct (Crous et al. 2015; Hernández-Restrepo et al. 2016a). A comparative synopsis of these genera is provided (Table 3).

**Table 3.** Synopsis of *Castanediella*-like genera.

Genera	Conidiophores	Conidiogenous cells	Conidia	Chlamydospores
* Castanediella *	Branched, pale brown to brown at the base and subhyaline at the apex.	Sympodial, small denticles or scars, subhyaline.	0–1-sepate, falcate, lunate, cylindrical or fusiform, hyaline	Not observed.
* Idriella *	Brown, mostly reduced to conidiogenous cells	Denticulate, sympodial	Aseptate, lunate, curved, hyaline	Brown, uni- or pluricellular.
* Idriellopsis *	Unbranched, brown at the base, almost hyaline at the apex, mostly reduced to conidiogenous cells	Terminal, denticulate	0–1-septate, falcate, curved, hyaline	Not observed
* Microdochium *	More or less verticillate, reduced to conidiogenous cells, hyaline	Hyaline, sympodial or percurrent, sometimes denticulate	Aseptate or multiseptate, lunate, falcate, fusiform, filiform, obovoid or subpyriform, straight or curved, hyaline	Terminal or intercalary, solitary, in chains or grouped in clusters, brown.
* Neoidriella *	Mostly unbranched, pale brown, mostly reduced to conidiogenous cells	Sympodial, denticulate, terminal.	Aseptate, cylindrical to obovoid, hyaline	Intercalary or terminal, pale brown.
* Paraidriella *	Unbranched, pale brown, mostly reduced to conidiogenous cells.	Sympodial, denticulate, terminal.	Aseptate, cylindrical to oblong, hyaline	Not observed.
* Selenodriella *	Unbranched or verticillate, brown.	Sympodial, denticulate, terminal and intercalary.	Aseptate, falcate, hyaline	Not observed

## Supplementary Material

XML Treatment for Castanediella brevis

XML Treatment for Castanediella monoseptata
